# Absorption of Dentine

**Published:** 1876-10

**Authors:** 


### ARTICLE VII.

### *Absorption of Dentine.*

Very recently two cases of absorption of the wall of the cavity between the plug and pulp have fallen under my observation, which I wish to make note in the Journal, particularly for the benefit of those dentists of long experience who have never seen, and therefore claim that such cases do not occur.

Dr. G., 38 years of age, complained of severe and continued pain in the 1st left superior bicuspid. An examination showed the tooth to contain two tin-foil fillings, distal and mesial, in an excellent state of preservation, although they had been inserted ten years previously. The tooth showed no signs of decay either above or around the plug neither was any other portion of the tooth attacked by caries. This was the first time that the tooth had ever given any trouble, but now the pain was so intense that the patient demanded extraction unless relief could be obtained instantly. I first removed the plug on the distal surface, thinking that perhaps there might possibly be a cavity above the plug. I found nothing of the kind, but on the contrary the cavity was as clean and free from discoloration and decay as if it had that moment been prepared for filling. I then removed the filling in the opposite side of the tooth, although from all external appearances one would not suspect that the source of trouble could be here.

This also was a tin foil plug, and one of the best and most dense tin fillings I ever saw. In order to remove it, I was obliged to cut and drill in every direction, thus proving by its density and other good qualities the skill of the operator, Dr. Pierce, now of Akron, O.

Upon the removal of this plug, which had faithfully guarded the tooth for years, the source of pain was discovered.

Although the wall of dentine protecting the pulp was as thick as a silver ten cent piece, still a small circular canal was plainly visible, connecting the now highly-inflamed pulp with the filling. An application of carbolic acid produced instantaneous relief, the first the patient had experienced for 18 hours. Arsenic was then applied, the pulp removed and the tooth again filled.

The other case of absorption occurred under a gold plug, 20 years old, but perfect in every particular. The patient was 45 years of age, and before coming to me, had suffered with his tooth for a week. It, like the first case, was excessively sensitive to heat or cold. I removed the plug and carefully syringed the cavity with tepid water, and then saw the living pulp fully exposed.

I treated this case as the other described, viz.: by destroying the pulp and filling roots and cavity of decay.

Why a tooth, after being filled and remaining perfectly quiet for 10 or 20 years, should, without any apparent cause, become affected in this manner, is a question that I will not undertake to solve. X. L.—*Misouri Dental Journal.*

